# Connexin-Mediated Signaling at the Immunological Synapse

**DOI:** 10.3390/ijms21103736

**Published:** 2020-05-25

**Authors:** Andrés Tittarelli, Mariela Navarrete, María Alejandra Gleisner, Peter Gebicke-Haerter, Flavio Salazar-Onfray

**Affiliations:** 1 Programa Institucional de Fomento a la Investigación, Desarrollo e Innovación (PIDi), Universidad Tecnológica Metropolitana (UTEM), Santiago 8940577, Chile; 2 Millennium Institute on Immunology and Immunotherapy, Faculty of Medicine, Universidad de Chile, Santiago 8380453, Chile; mariela.navarrete.s@gmail.com (M.N.); alejandra.gleisner@gmail.com (M.A.G.); Peter.Gebicke-Haerter@zi-mannheim.de (P.G.-H.); 3 Disciplinary Program of Immunology, Institute of Biomedical Sciences, Faculty of Medicine, Universidad de Chile, Santiago 8380453, Chile; 4 Institute of Psychopharmacology, Central Institute of Mental Health, Faculty of Medicine, University of Heidelberg, J5 68159 Mannheim, Germany

**Keywords:** connexin-43, gap junction, immunological synapse, signaling, cytotoxic immunological synapse

## Abstract

The immunological synapse (IS) is an intercellular communication platform, organized at the contact site of two adjacent cells, where at least one is an immune cell. Functional IS formation is fundamental for the modulation of the most relevant immune system activities, such as T cell activation by antigen presenting cells and T cell/natural killer (NK) cell-mediated target cell (infected or cancer) killing. Extensive evidence suggests that connexins, in particular connexin-43 (Cx43) hemichannels and/or gap junctions, regulate signaling events in different types of IS. Although the underlying mechanisms are not fully understood, the current evidence suggests that Cx43 channels could act as facilitators for calcium ions, cyclic adenosine monophosphate, and/or adenosine triphosphate uptake and/or release at the interface of interacting cells. These second messengers have relevant roles in the IS signaling during dendritic cell-mediated T and NK cell activation, regulatory T cell-mediated immune suppression, and cytotoxic T lymphocyte or NK cell-mediated target tumor cell killing. Additionally, as the cytoplasmic C-terminus domain of Cx43 interacts with a plethora of proteins, Cx43 may act as scaffolds for integration of various regulatory proteins at the IS, as suggested by the high number of Cx43-interacting proteins that translocate at these cell-cell interface domains. In this review, we provide an updated overview and analysis on the role and possible underlying mechanisms of Cx43 in IS signaling.

## 1. Introduction

The immunological synapse (IS) is a specialized contact area formed between two adjacent cells, where at least one of them is an immune cell. This cell contact structure is characterized by a close apposition of an immune cell membrane with the membrane of an adjacent cell, induced by adaptive or innate immune recognition, intercellular adhesion, stability and polarized signaling.

The formation of a functional IS is fundamental for the modulation of most relevant immune system activities, such as the priming and activation of T (cytotoxic CD8^+^ and helper CD4^+^) and natural killer (NK) cells by professional antigen presenting cells (APCs), like dendritic cells (DC), macrophages, and B cells [1,2]; killing of target (infected or cancer) cells by NK cells and cytotoxic T lymphocytes (CTL), via the formation of a cytotoxic IS (CIS) [3]; phagocytosis of microbes by myeloid phagocytes [4]; inflammatory responses mediated by mast cells via an antibody-dependent degranulatory synapse [5]; antigen extraction, processing and presentation by B cells [6]; and regulatory T cell (T_reg_)-mediated immune suppression [7].

**Figure 1:**
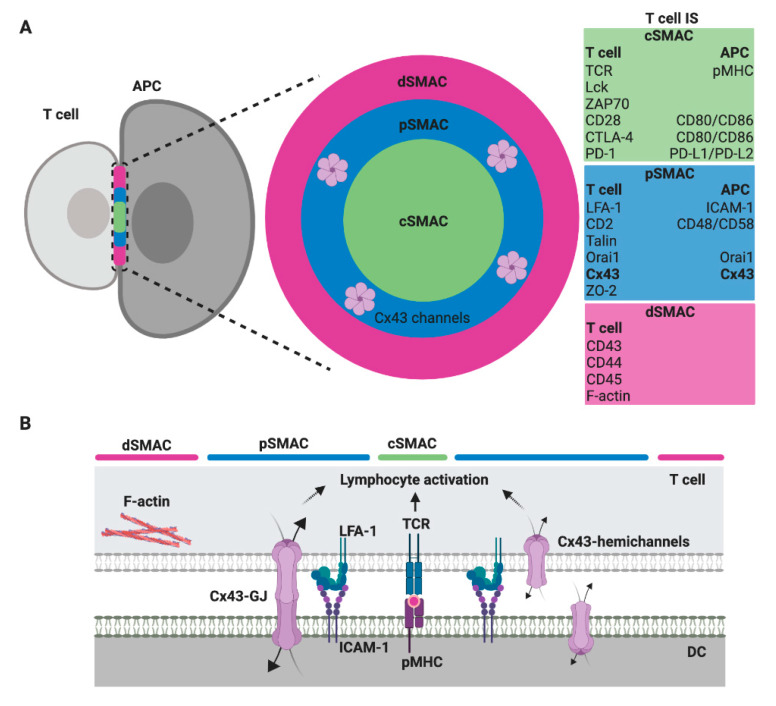
Scheme of a T cell immunological synapse (IS) and localization of Cx43 formed gap junctions (GJ) in the SMAC. (**A**) A face on view of the IS with the characteristic SMAC patterns, including the cSMAC (green), the pSMAC ring surrounding the cSMAC (blue) and the distal region to the synapse outside the pSMAC (dSMAC, red), as well as the molecules/ligand that are found enriched within. The evidence suggests that gap junction (GJ) channels formed by Cx43 (Cx43-GJ), as well as Cx43 hemichannels, are located in the pSMAC region [13]. (**B**) A profile view showing a selection of key ligand pairs and Cx43 channels (GJ and hemichannels) that are involved in DC-mediated T cell activation.

Regardless of the type of interacting immune cell, a mature IS comprises highly ordered and plastic signaling platforms that integrate signals and coordinates molecular interactions leading to appropriate immune responses [8]. These signaling platforms are organized in at least three concentric regions called supramolecular activation clusters (SMAC): the central, the peripheral and the distal SMAC (cSMAC, pSMAC and dSMAC, respectively) [9,10]. These organized structures are more characteristic of T and B cell IS, but some of these molecular organizations are also found in the CIS from NK cells [11]. In general, the cSMAC, a molecular platform that mediates both proximal signaling events and active secretion, is organized as a cluster of T cell receptor (TCR), B cell receptor (BCR) or activating/inhibitory NK cell receptors, associated signaling molecules, co-stimulatory receptor/ligands, and a secretory domain. The pSMAC includes adhesion molecule interactions, like lymphocyte function-associated antigen-1 (LFA-1)/intercellular adhesion molecule-I (ICAM-1), which promote the stable adhesion of interacting cells; whereas a ring of filamentous actin (F-actin), which exerts mechanical forces required for IS activity, is generally accumulated at the dSMAC (Figure 1) [9,10,12].

Gap junctions (GJ) are clusters of intercellular channels found at the plasma membrane of interacting cells that allow its direct communication. Each GJ is formed by two connexons, which are hexameric hemichannels of connexin (Cx) proteins inserted into the plasma membrane of the cells, each one provided by each of the two contacting cells [14]. These Cx-formed hemichannels can also work as uncoupled channels, allowing the transfer of chemical information from the cytoplasm to the extracellular milieu, and vice versa. Once functional Cx-channels are established, they allow the bidirectional transfer of small molecules (up to ≈1.4 nm) of varied nature, including adenosine triphosphate (ATP), cyclic adenosine monophosphate (cAMP), inositol triphosphate (IP_3_), calcium, small peptides (including antigens), and microRNAs [15]. There are 20 Cx members in mice and 21 in humans, and the different isoforms determine channel properties. Cxs are generally expressed in a tissue-specific manner, with the exception of Cx43, that is expressed almost ubiquitously and is the main Cx member expressed in the immune system cells [16]. Practically all the immune cells and their hematopoietic precursors express Cx43, and nowadays, its participation in the modulation of different aspects of immune responses is well recognized, as we and other groups recently reviewed [17,18]. Other Cx isoforms like Cx45, Cx40, Cx37, Cx30.3, and Cx26 have also been implicated in inflammatory or other immunological events [19,20,21,22]. However, until now, a majority of accumulated data in immunological literature refers to Cx43, probably due to a higher development of molecular tools, such as specific antibodies and inhibitors.

Eighteen years have passed since Oviedo-Orta and Evans suggested, for the first time, a role for GJ and Cx as potential contributors to the IS signaling [23]. Since then, an important number of experimental evidence have been generated on Cx43-mediating signaling at the IS, which are summarized below.

## 2. Cx43-Mediated Signaling at the DC-T Cell IS

**Figure 2:**
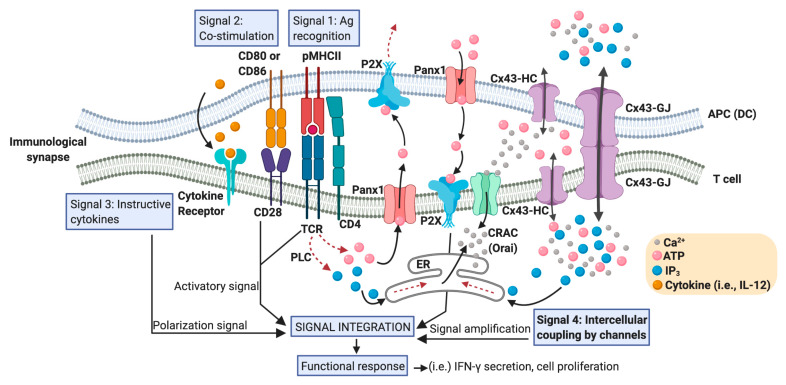
Positioning Cx43 in the intercellular coupling by channels in the DC-T cell IS. An IS between an APC (particularly a DC) and a T cell that effectively promotes T cell activation contains a large number of signaling molecules and signaling events that converge in order to generate T cell functional responses. These signaling events include the activation signal produced by TCR-pMHC ligation (antigen recognition: signal 1), plus co-stimulatory receptor engagement (signal 2), and a polarization signal yielded by instructive cytokines released by the APC (signal 3). Calcium release activated calcium channels (CRAC), such as Orai; hemichannels such as those formed by Panx1 and Cx43; and Cx43 formed gap junctions (Cx43-GJs), are involved in the Ca^2+^- and ATP- (via purinergic P2X receptors) mediated signaling amplification required for antigen-dependent T cell activation. Although Ca^2+^ can diffuse through Cx43 channels, the current consensus is that inositol triphosphate (IP_3_) is the critical intercellular signal that modulates Ca^2+^ release from internal cellular stores, like endoplasmic reticulum (ER), and it is responsible for GJ-mediated Ca^2+^ waves in other cell models. An increase in the levels of IP_3_ in T cells could be produced by the convergence of those generated by phospholipase C (PLC) activation and those acquired from DC via Cx43 (or other Cx isoform) channel-mediated communication. We called these signaling events ‘intercellular coupling by channels’ and considered them as signal 4 in a DC-T cell IS. In response to TCR and CD28 stimulation, Panx1, purinergic P2X receptors, CRAC, and Cx43 translocate to the IS. ATP released through hemichannels promotes autocrine/paracrine signaling via the P2X receptors and the entry of Ca^2+^ into T cell. Curved arrows indicate movement of molecules; dotted arrows indicate a signaling pathway; straight arrows connect concepts.

An efficient T cell-mediated adaptive immunity relies on the ability of these cells to differentiate and to make a concerted response. Therefore, an immune response requires that specific T cells find a cognate antigenic peptide complexed to major histocompatibility complex (MHC) molecules (pMHC) on the surface of APCs, and receive appropriate signals to differentiate into effector (T_eff_), regulatory or memory subsets. These fundamental signals are sensed by the T cell during the establishment of an IS with the DC, and are typically categorized in three different types: signal 1, which is supported by antigen recognition (TCR-pMHC); signal 2, corresponding to the co-stimulation given by the interaction of CD28-CD80/CD86, CD40-CD40L, ICOS-ICOSL, among others; and signal 3, which is an instructive cytokine-driven signal [24] (Figure 2). Additional upstream signaling events are also fundamental for T cell priming and the polarization of T cell-mediated responses. These signals are related to how DCs were activated and their migration induced by pattern recognition receptors ligation by pathogen-associated molecular patterns and/or danger-associated molecular patterns. These molecular events have been designed as the signal 0, and provoke the start of an immune response [25]. Nevertheless, even more upstream, the activation of cell death pathways on infected or tumor cells, from where the cognate antigens derived, has been proposed as an initiating immunological event, resulting in the generation of the signal −1 [26]. Moreover, additional signaling pathways downstream of TCR activation modulate the strength and nature of the DC-mediated T cell activation. We termed one of these events as “intercellular coupling by channels” and we consider it as signal 4 (Figure 2). As we described below, these mechanisms are of extreme relevance in the signaling amplification induced by pMHC-TCR interactions.

Recent evidences suggest that purinergic signaling, together with the global and local accumulation of Ca^2+^ at the IS proximity, serve as the signal amplification mechanisms needed for T cell activation driven by antigen recognition [27,28,29]. The intercellular coupling by Cx- and pannexin (Panx)-formed channels could have a fundamental role in these processes. Panxs are a family of proteins similar to Cxs, consisting of three members, Panx 1, 2, and 3 [30]. Panxs can form plasma membrane hemichannels, but unlike Cxs, do not produce canonical GJs [31].

T cells and DCs express many members of the purinergic receptor families (P2X, P2Y and P1) [27], and can release ATP in response to various extracellular stimuli, including antigen-specific interactions [32,33,34,35,36]. Upon TCR engagement with anti-CD3/CD28 antibody-coated beads, T cells use Panx1- and Cx-formed hemichannels to release ATP, resulting in the subsequent activation of purinergic receptors P2Xs, thus facilitating both the influx of extracellular Ca^2+^ and the expression of effector cytokines [32,35,36]. Interestingly, TCR stimulation with anti-CD3/CD28 antibody-coated beads also results in the polarization of P2X1, P2X4 receptors, calcium channels (STIM1 and Orai1), and Panx1 hemichannels to the IS [33]. The inhibition of hemichannels with Panx1-specific mimetic peptides or by carbenoxolone, which is a chemical inhibitor of both Panx- and Cx-formed channels, suppresses TCR-induced ATP release, Ca^2+^ entry and T cell activation [33], indicating that purinergic signaling plays an active role at the IS in DC-mediated T cell activation.

Upon activation, Cx43 expression, both at mRNA and protein level, increases in DCs and T cells [15,18,37,38,39], suggesting that these cells acquire the capacity to interact with each other through Cx43 channels in order to modulate adaptive immune responses. In this sense, Elgueta and coworkers showed the formation of functional GJ between murine splenic or bone marrow-derived DCs with CD4^+^ and CD8^+^ T cells [40]. In their studies, they used two different TCR-transgenic antigenic models: pigeon cytochrome-c peptide presented in the context of I-E^k^ molecules, and ovalbumin peptides presented in H2-K^b^ and I-A^b^ molecules. The formation of GJ-mediated coupling between DC and T cells was sensitive to chemical inhibitors of GJs as oleamide and, more importantly, to Cx43-mimetic blocking peptides [40]. The inhibition of GJs reduced DC-mediated T cell activation, reflected by lower T cell proliferation, CD69 expression and IL-2 secretion. Interestingly, the authors demonstrated that Cx43-GJ blockers did not affect the polyclonal activation of CD4^+^ T cells induced with soluble anti-CD3/anti-CD28 antibodies in the absence of DCs, indicating that the inhibition of Cx43 channels inhibit T cell activation by directly interfering with GJ assembly between DCs and T cells. Of note, this last experiment differs from those described in the previous paragraph (references 31, 32, 34), in that TCR activation was performed with soluble instead of bead-coated-antibodies. Whereas beads promote the formation of an IS-like structure in the T cells, soluble antibodies do not [41], thus reinforcing the idea of the role of synaptic Cx43 channels in the T cell activation.

In this context, we reported that both hemichannels and GJ formed by Cx43 (Cx43-GJ), polarize to the DC-T cell IS in an antigen- and actin cytoskeleton-dependent manner in murine and human cell models [13]. Indeed, we described that Cx43 channels accumulate preferentially in the pSMAC, colocalizing with LFA-1 molecules (Figure 1). Using Cx43-specific inhibitors, we also showed that Cx43-mediated intercellular communication between DC and T cells is bidirectional [13]. Although the nature of the molecules passing by Cx43 channels in the DC-T cell IS was not fully determined, the silencing of Cx43 expression (either on DCs or T cells) or the inhibition of Cx43-GJ docking, strongly impaired both IFN-γ secretion and the increase of intracellular Ca^2+^ in the T cells interacting with antigen-loaded DCs [13]. These results strongly suggest that IS-located Cx43 channels participate, directly (as the Orai1 calcium channels) or indirectly, via the release of ATP (as Panx1 hemichannels) and/or through the uptake of IP_3_ from DC (as described in other cell models [42]), in the Ca^2+^ elevation required for signaling amplification and T cell activation (Figure 2).

In addition to its role in DC-mediated activation of T_eff_ cells, Cx43 channels also participate in the modulation of the regulatory activity of CD4^+^ T_regs_. T_reg_ cells, which are fundamental players in immune homeostasis and protection against autoimmunity, mediate their suppressive action by acting directly on T_eff_ cells or DCs, through cell contact-dependent and -independent mechanisms. Among the cell contact-dependent mechanisms, the Cx43-GJ-mediated transfer of cAMP is one of the most relevant. T_reg_ and T_eff_ cells showed differential expression and activation of enzymes that regulate intracellular cAMP levels, which translates in T_regs_ having higher levels of cAMP than T_eff_ cells [43,44]. In 2007, Bopp and coworkers showed that naturally occurring T_regs_ inhibit CD4^+^ T_eff_ proliferation, CD69 expression and IL-2 production through the transfer of cAMP via Cx43-GJ-mediated intercellular communications, in vitro as well as in vivo [44]. This second messenger, once inside the cell, triggers different downstream signaling cascades, leading to its immune regulation [45]. In agreement with data showing that DCs are the primary target of T_reg_ cell-mediated suppression [46], the amount of cAMP transferred via Cx43-GJs from T_regs_ to DCs is significantly higher than that transferred to T_eff_ cells [47]. Indeed, the immune regulation of DCs by cAMP transferred from T_regs_ via Cx43-GJs has been proposed as a mechanism for controlling graft-versus-host disease after allogeneic hematopoietic stem cell transplantation [47]. Additionally, it has been shown that the reduced suppressive potency of T_reg_ cells of non-obese diabetic (NOD) mice is due to its impaired Cx43 expression and lower capacity to form GJ channels [48]. Indeed, Cx43 overexpression or the strengthening of Cx43-GJ-mediated intercellular communications using the α-Cx carboxyl-terminal synthetic Cx43 mimic peptide 1 (αCT-1), which enhances GJ aggregation by disrupting the interaction between Cx43 and zonula occludens (ZO)-1 [49], increases the suppressive properties of these NOD-derived T_reg_ cells [48]. GJ-mediated coupling between T_reg_ cells and lymph node-resident DCs has also been observed during the induction of tolerance to low doses of allergens in mice [50]. Moreover, T_reg_ cells also abrogate the de novo induction of hapten-specific CD8^+^ T cell-driven immune reactions, by interfering with T cell stimulatory activity of DCs through Cx43-GJ intercellular communication [51]. Importantly, Cx43-GJ blockage between T_regs_ and T_eff_ cells abolished T_reg_ cell-mediated suppression of human immunodeficiency virus replication, indicating that Cx43 channels also have an important impact in the outcome of this viral infection [52].

## 3. Cx43 Channels also Impact DC-Mediated T Cell Activation by Amplifying Antigen Cross-Presentation Pathways

The involvement of Cx43 channels in DC-mediated T cell activation is not only limited to its role in facilitating signaling amplification at the IS, but also in DC maturation [53], DC migration to lymph nodes [54] and, as we described below, in promoting the spreading and amplification of antigen cross-presentation pathways.

In 2005, Niejssen and coworkers showed, for the first time, that peptides with a molecular mass of up to approximately 1800 Daltons can diffuse through GJs from the cytoplasm of an virus-infected cell to the cytoplasm to an APC for its cross-presentation, causing cytotoxic T-cell recognition of adjacent, innocent bystander cells [55]. Our group has shown that TNFα-stimulated human DCs pulsed with a melanoma cell lysate can establish functional GJ-mediated intercellular communications and promote melanoma antigen transfer between ex vivo produced DCs. The use of GJ and Cx43 inhibitors suppressed the antigen transfer between DCs and therefore reduces melanoma-specific T cell activation [37]. Additionally, Cx43-GJs have also been implicated in the antigenic peptide transfer from melanoma to autologous endothelial cells. Once endothelial cells acquire the melanoma antigens, they become susceptible to cross-recognition and elimination by autologous tumor-specific CTLs [56].

It is worth noting that Cx expression by cancer cells is generally low or null, therefore this Cx-dependent mechanism of antigen acquisition for cross-presentation is altered in most tumors, leading to a lack of tumor-specific T cell activation, and therefore a weak anti-tumor response. In this context, Saccheri and coworkers showed that infection with Salmonella can induce the up-regulation of Cx43 in murine melanoma cells, leading to the establishment of functional GJs with adjacent APCs, transfer of pre-processed tumor antigens and the consequent cross-presentation in their surface by MHCI [57]. These pMHCI thus activate cytotoxic T cells against the tumor antigens, which could efficiently control the growth of distant uninfected tumors. The Cx43 silencing in bacteria-infected melanoma cells leads to the failure to elicit a cytotoxic antitumor response, suggesting that this mechanism of cross-presentation is the principal one used in vivo for the generation of antitumor responses [57]. Moreover, DC ex vivo loaded with Salmonella-infected melanoma cells, shows higher efficiency in inducing tumor growth inhibition compared to other types of DC approaches, depending on Cx43 expression in the melanoma cells used as an antigen source [57]. These results strongly suggest that the acquisition of antigenic peptides by DCs via Cx43-GJ-mediated communications with tumor cells is far more effective than standard pathways for antigen loading during the generation of protective DC vaccines. In this regard, recently, it has been shown that Cx43-mediated antigen transfer impact the efficacy of APC-based cancer vaccines, where the activation of T cells requires the priming activity of endogenous DCs, which are activated through the transfer of peptides via Cx43-GJs from ex vivo loaded monocytes to endogenous CD8^+^ splenic DCs [58]. The formal demonstration for the existence of a Cx43-mediated antigenic peptide transfer pathway is highly relevant for the design of successful anti-cancer immunotherapy treatments [58]. Interestingly, DCs can also acquire tumor antigens from apoptotic tumor cells via a direct GJ-mediated mechanism [59]. Given that caspase activation can expose neo-epitopes in apoptotic tumor cells through the direct cleavage of target proteins, this mechanism could increase the repertoire of cross-presented antigens, and therefore be of great relevance in tumor immunity.

A similar pathway of antigen cross-presentation has also been found in the generation of oral tolerance to antigens [60]. Mainly, gut resident macrophages acquire soluble antigens and transfer them through Cx43-formed GJs to CD103^+^ DCs. The deletion of Cx43 in DCs impairs this antigen transfer and results in the incapacity of DCs to acquire and present fed antigens to T cells, leading to a diminished T_reg_ cell differentiation and lower immune tolerance, suggesting a physiological role of GJs in oral acquired antigen presentation [60].

## 4. Cx43-Mediated Signaling at the CIS

**Figure 3:**
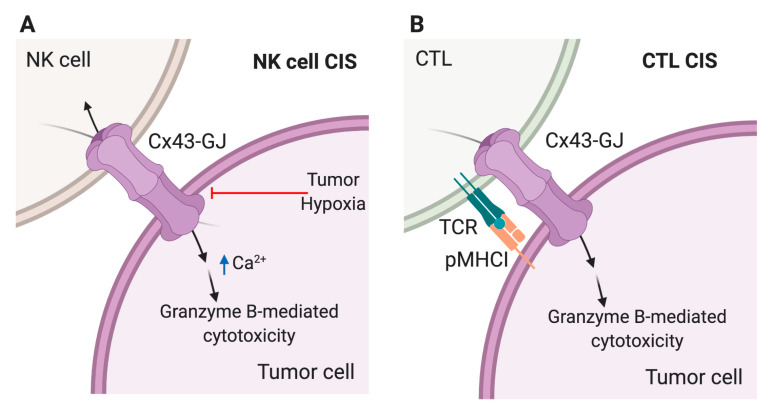
Cx43-mediated signaling at the cytotoxic immunological synapses (CIS). Cx43 gap junction (Cx43-GJ) channels polarize to the CIS formed during NK cell (A) and CTL (B) mediated killing of tumor cells. (**A**) NK cells and target tumor cells form bidirectional GJ coupling (represented by the two arrows crossing the Cx43-GJ) that allow, via an unknown mechanism, an efficient Ca^2+^ influx into the target cells, contributing to granzyme B activity and apoptosis of tumor cells (indicated by the arrow). Hypoxia induces the degradation of Cx43-GJ and renders hypoxic tumor cells resistant to NK cell-mediated cytotoxicity (indicated by the T-bar red arrow). (**B**) Cx43-GJ formation and coupling (indicated by the arrow crossing the Cx43-GJ) is necessary for an efficient antigen-dependent CTL-mediated tumor cell lysis (represented by the arrow).

CTLs and NK cells establish a regulated and organized supramolecular structure with similar features to the DC-T cell IS, called CIS. The assembly of the CIS allows effector cells to kill target tumor or infected cells in a specific manner, without affecting the neighbor healthy, non-malignant cells [11]. The arrangement of the CIS at a specific contact point with target cells, assures the polarized release of cytotoxic granules, containing mainly granzyme B and perforin, to malignant or infected cells, activating an apoptotic cascade within them. Previously, our group has reported that Cx43 polarizes to the CIS formed between NK cells and tumor cells, mediating bidirectional communications during the NK cell cytotoxic process [61] (Figure 3A).

NK cells and target tumor cells form bidirectional GJ coupling, which is effectively reduced by blocking Cx43. Cx43-GJ intercellular communications among NK cells and tumor cells appear to not affect tumor-induced NK-cell degranulation, but to regulate an efficient Ca^2+^ influx into the target cells, contributing to granzyme B activity and therefore leading to apoptosis (Figure 3A) [61]. The influx of Ca^2+^ in target cells was reported as necessary for both NK cell- and CTL-mediated granzyme B-induced apoptosis [62,63,64]. The formation of gigantosomes (large endosomes) inside the target cells is a Ca^2+^-dependent process, in which granzyme and perforin are endocyted near the CIS. It has been shown that pores formation in plasma membrane caused by perforin is essential for Ca^2+^ influx into target cells [62,63,64], suggesting that Cx43 could cooperate with these pores in the induction of required Ca^2+^ influx, responsible for the gigantosome formation and/or contributing to the release of granules cytotoxic content, leading to granzyme B activity inside the target cells.

In fact, the ectopic expression of Cx43 in target tumor cells makes them more susceptible to NK cell killing, while the blocking of GJ coupling by chemical inhibitors or Cx43 mimetic peptides reduces the susceptibility of tumor target cells to NK cell killing [65]. Moreover, increased expression of Cx43 due to hypoxic stress has been described in melanoma cells, which is promoted by the transcriptional activity of hypoxia-inducible factor-1α [65]. Despite that, hypoxic melanoma cells are less susceptible to NK cell-mediated lysis than Cx43-expressing melanoma cells cultured in normoxia. This was due to the fact that the CIS formed between NK and normoxic melanoma cells was more stable and contained a higher level of Cx43-forming GJs, whereas the synapse formed with hypoxic cells was less stable and contained a significant lower level of these Cx43 channels (Figure 3A). This phenomenon was due to autophagy activation in hypoxic melanoma cells, that selectively degraded gap-junctional Cx43, destabilizing the CIS and leading to the impairment of NK cell-mediated killing [65]. Additionally, we previously demonstrated that Cx43 polarizes at the contact site between CTLs and target melanoma cells in an antigen-dependent manner, allowing GJ-mediated communications between these cells during the CIS establishment [66] (Figure 3B). Knocking down or blocking Cx43 channels with specific siRNAs or mimetic peptides led to a decrease in the intercellular communication between CTLs and tumor cells, subsequently producing a diminished granzyme B activity within the target cells, suggesting that Cx43-GJ formation is necessary for an efficient CTL-mediated tumor cell lysis [66].

On the other hand, it has been observed that the CIS formed by chimeric antigen receptors (CAR)-T cells are more stable, potent and less reliant on LFA-1, generating stronger conjugates than the TCR-mediated CIS [67]. Interestingly, it was observed that Cx43 were enriched in CAR-T cell IS signaling, which are stronger and formed more rapidly than TCR CIS, suggesting that Cx43 could play an essential role in the formation of an effective, adequate CIS, even in the presence of disorganized LFA-1 adhesion rings [67].

## 5. Cx43 Interactome Reveals Multiple Proteins Associated with the IS

**Figure 4:**
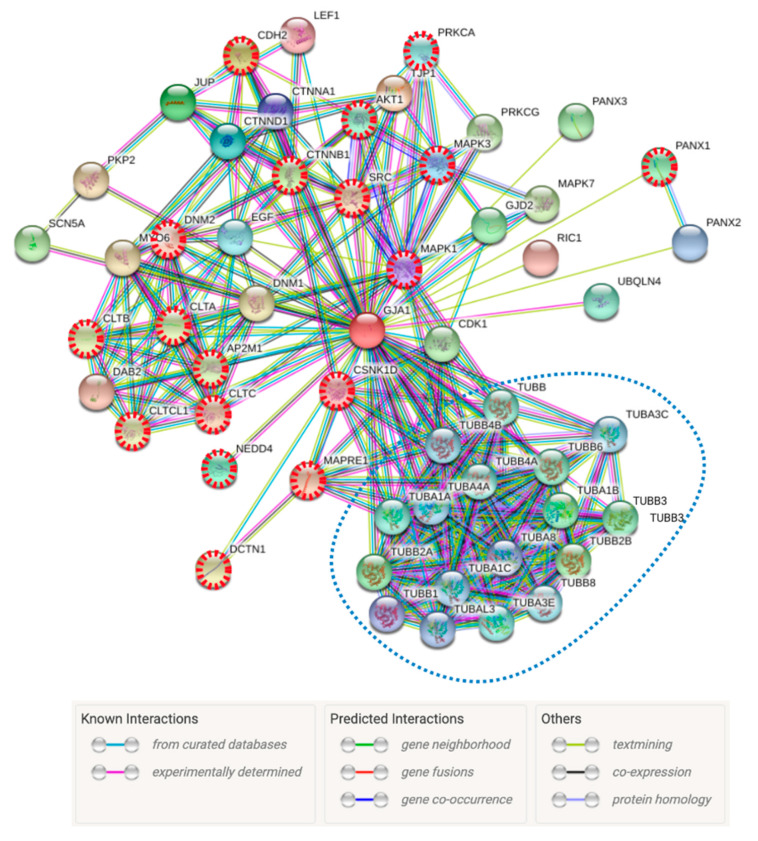
The Cx43 (GJA1) interactome is enriched with proteins associated with the IS. STRING analysis of the most prevalent Cx43 (GJA1) interacting proteins. Nodes with red non-continuous red lines are proteins with published evidence that strongly associate them with the IS (18 proteins). Included in the non-continuous blue line circle are highlighted the cluster containing 17 tubulins. The network was retrieved and constructed using the STRING database version 11.0, using a stringent confidence score prediction setting (>0.8), resulting in 54 interactions. Full names, score prediction and details of the top 25- interactions are shown in Table 1.

Cx43 not only contributes to the formation of intercellular channels that allow the direct communication between cells, but it also acts as a scaffold molecule for cell signaling networks, given its extensive protein interactome [68]. This characteristic of Cx43 is conferred by its post-translational modifiable C-terminus cytosolic domain, which modulates the channel gating properties and the intercellular trafficking. Moreover, this C-terminus contains multiple protein interaction domains that allow crosstalk between Cx43 and other regulatory and cytoskeletal proteins [68]. For example, the Cx43 C-terminus domain is required for spreading and adhesion of B cells to endothelial cells via a LFA-1 and CXCL12-mediated Rap1 activation mechanism [69]. Therefore, we hypothesized that plasma membrane located Cx43 channels may also regulate, directly or indirectly, the recruitment, stabilization and/or turnover of additional molecules at the IS. Indeed, 15 of the top-25 Cx43 (GJA1)-interacting proteins (60%) have been directly associated with the IS (Table 1 and Figure 4). In addition to the proteins described in Table 1, Cx43 interacts with 17 different tubulins (Figure 4), with a very well known role in the sailing and docking at the IS [70]. On the other hand, the interacting protein with the highest score is TJP1 (Table 1), also known as ZO-1. It has been reported that Cx43 interacts with ZO-1 and ZO-2 (TJP2) [71]. The interaction of Cx43 with ZO-1 regulates the rate of undocked Cx43 hemichannel aggregation into GJs [49]. The participation of ZO-2 in the IS established among T cells and APCs has recently been described [72], suggesting that ZO-2 could participate in GJ formation at the IS. Altogether, this evidence indicates that the study of Cx43 interactome at the IS deserves further attention.

## 6. Concluding Remarks

Cx43-mediated intercellular communications have an important role in different aspects of immunity [16], including during the formation of IS. Cx43 hemichannels and/or GJs have been proved to regulate some signaling events in different IS, such as those formed between DC with T and NK cells, CTL and NK cells with tumor target cells, or T_reg_ cells with DCs or T_eff_ cells. Although the mechanisms involved in the role of Cx43 in these IS are not fully understood, current evidence suggests that Cx43 channels could act as facilitators for Ca^2+^, cAMP, and/or ATP uptake and/or release at the interface of the interacting cells. These second messengers have relevant roles in the IS signaling during APC-mediated T cell and NK cell activation, T_reg_-mediated immune suppression, and CTL or NK cell-mediated target cell killing. Moreover, as the intracellular C-terminus domain of Cx43 interacts with a plethora of proteins, Cx43 may also act as a scaffold for the integration of different signaling events, as suggested by the great number of Cx43-interacting proteins that also translocate at the IS. The emerging understanding regarding the role of Cx43 channels in IS signaling could provide important insight contributing to the development of novel therapeutic approaches to modulate immune responses in different pathologies, like enhancing T cell mediated immunity against cancer or regulating graft rejection in transplantation.

## Figures and Tables

**Table 1:** The top-25 Cx43 protein interactome and its association with the IS. The top-25 (highest prediction setting of (scores >0.9)) interactome for Cx43 was retrieved and constructed using the STRING database version 11.0 (http://string-db.org). In bold letters and gray boxes are highlighted proteins that, like Cx43, translocate to and modulate the function of IS, and its associated reference.

Protein	Annotation	Score	Reference
TJP1	Tight junction protein-1 (zonula occludens-1; ZO-1).	0.994	
**CDH2**	Cadherin 2, type 1, N-cadherin. Neuroimmune synapses with mast cells that involve N-cadherin expression on the mast cells may be important in allergy.	0.99	[73]
**CTNNB1**	Catenin (cadherin-associated protein), beta 1. Key downstream component of the canonical Wnt signaling pathway. Serves as anchor protein at actin-rich adherent junctions and at the IS.	0.964	[74]
JUP	Junction plakoglobin. Catenin (cadherin-associated protein), gamma.	0.947	
**NEDD4**	E3 ubiquitin-protein ligase. Involved in the proteolytic degradation of IS signaling proteins (PLC-γ1 and PKC-θ), from T cell-APC contact membrane in T cell anergy.	0.947	[75]
CTNND1	Catenin (cadherin-associated protein), delta 1.	0.927	
**MAPK3**	Mitogen-activated protein kinase 3 (ERK1). Serine/threonine kinase which acts as an essential component of the MAP kinase signal transduction pathway. Mediates the signaling of lamin-A-dependent F-actin-mediated IS formation and T cell activation. Mediates LFA-1-dependent TCR activation in CD8^+^ T cells.	0.938	[76,77]
CTNNA1	Catenin (cadherin-associated protein), alpha 1.	0.934	
**MAPK1**	Mitogen-activated protein kinase 1 (ERK2). Serine/threonine kinase which acts as an essential component of the MAP kinase signal transduction pathway. Mediates the signaling of lamin-A-dependent F-actin-mediated IS formation and T cell activation.	0.985	[76,77]
LEF1	Lymphoid enhancer-binding factor 1.	0.922	
**CSNK1D**	Casein kinase 1, delta (CKIδ). Phosphorylates Cx43/GJA1, MAP1A, SNAPIN, MAPT/TAU, TOP2A, DCK, HIF1A, EIF6, p53/TP53, DVL2, DVL3, ESR1, AIB1/NCOA3, DNMT1, PKD2, YAP1, PER1 and PER2. Controls centrosome recruitment to the IS during T cell activation.	0.922	[78]
RIC1	RAB6A-GEF complex partner protein 1. Required for phosphorylation and localization of Cx43.	0.922	
DAB2	Disabled homolog 2; Adapter protein that functions as clathrin-associated sorting protein required for clathrin-mediated endocytosis of selected cargo proteins.	0.92	
**DNM2**	Dynamin-2. Regulates T cell activation by controlling actin polymerization at the IS.	0.919	[79]
**MAPRE1**	Microtubule-associated protein, RP/EB family, member 1 (EB1). Binds to the microtubules plus-end and regulates dynamics of microtubule cytoskeleton. CKIδ/EB1 contributes to the increase in microtubule growth speeds in polarized T cells and to centrosome recruitment to the IS during T cell activation. Mediates the organization of an IS fully functional to transduce activation signals.	0.916	[78,80]
**CLTC**	Clathrin heavy chain 1. Clathrin is recruited to the IS and drives actin cytoskeleton accumulation. Promotes endocytosis of cytotoxic granules in target cells during in CIS.	0.91	[81,82]
**SRC**	v-src sarcoma (Schmidt-Ruppin A-2) viral oncogene homolog (avian). Non-receptor protein tyrosine kinase which is activated following engagement of many different classes of cellular receptors including immune response receptors and integrins. Src is activated early during IS formation.	0.909	[83]
PKP2	Plakophilin 2.	0.908	
MYO6	Unconventional myosin-VI.	0.906	
DNM1	Dynamin-1.	0.905	
**DCTN1**	Dynactin 1. Mediates the accumulation of CTLA4 and granzyme B-containing intracellular vesicles at the IS and CTL-mediated lysis. Dynein/dynactin contribute to the internal forces that control organelle positioning and function at the T cell-APC contact area.	0.905	[70,84]
**CLTCL1**	Clathrin heavy chain 2. Clathrin is recruited to the IS and drives actin cytoskeleton accumulation. Promotes endocytosis of cytotoxic granules in target cells during in CIS.	0.902	[81,82]
**CLTB**	Clathrin light chain B. Clathrin is recruited to the IS and drives actin cytoskeleton accumulation. Promotes endocytosis of cytotoxic granules in target cells during in CIS.	0.902	[81,82]
**CLTA**	Clathrin light chain A. Clathrin is recruited to the IS and drives actin cytoskeleton accumulation. Promotes endocytosis of cytotoxic granules in target cells during in CIS.	0.902	[81,82]
**AP2M1**	AP-2 complex subunit mu. Involved in clathrin-dependent endocytosis in which cargo proteins are incorporated into vesicles surrounded by clathrin, which are destined for fusion with the early endosome. Participates in TCR recycling at the IS.	0.9	[82]
